# Immune Checkpoint Inhibitors and Platelets from Treatment-Induced Thrombocytopenia to Complex Immune and Immune-Related Adverse Events

**DOI:** 10.3390/cancers18172773

**Published:** 2026-08-26

**Authors:** Árpád Illés, Miklós Udvardy, Zsófia Miltényi

**Affiliations:** 1 Department of Haematology, Faculty of Medicine, University of Debrecen, Member of ERN-EuroBloodNet (European Reference Network on Rare Haematological Diseases), 4032 Debrecen, Hungary; 2 Doctoral School of Clinical Medicine, University of Debrecen, 4032 Debrecen, Hungary

**Keywords:** immune checkpoint inhibitors, platelet, function, efficiency

## Abstract

Immune checkpoint inhibitor-induced immune thrombocytopenia (ICI-ITP) is a rare but severe adverse event that may lead to bleeding complications and may require therapeutic changes or interventions. The clinical condition and interventions are highly complex; there are certainly tumour-type and combination-therapy differences, as well as individual ICI-agent factors that modify the risk of thrombocytopenia. ICI-ITP shares some common features, even though its pathogenesis differs markedly from that of traditional ITP. However, ICI-ITP is a clinically important complication; a deeper analysis of factors related to ICI–platelet interactions is also necessary. White blood cell–lymphocyte and platelet count ratios may have prognostic value in predicting the development of a low platelet count. Platelet counts themselves might influence the effectiveness of ICI-type anticancer interventions by facilitating T-cell-induced neoexpression of PD-1 on platelet surfaces. Baseline platelet counts and immunoglobulin levels may also modify treatment outcomes. Platelet activation can also promote immune-related adverse events. ICI-induced ITP-like syndrome’s prognostic factors are not only experimental facts but also clinically important. The connections among platelet counts, activation, immunoglobulins, and cell ratios are likely important factors influencing ICI-induced ITP risk reduction and the improvement of tumour-directed immune response by tailoring ICI selection to tumour type and specific baseline cellular characteristics.

## Simple Summary

This review aims to summarise comprehensive and concise information on changes in platelet count and function induced by checkpoint inhibitors and related immunomodulatory antitumour agents. Clinical conditions and interventions are highly complex; numerous factors—such as tumour type, ICI type, and combination therapies—modify the risk of thrombocytopenia. Several prognostic factors can predict its onset and influence treatment efficacy and survival, which is of great importance in everyday clinical practice.

## 1. Introduction

Since the advent of pioneering ICI-based antitumour immune therapies, platelet counts and functions are receiving more and more attention. As platelet activation, thrombocytopenia of myelosuppressive origin, traditional immune mechanisms, and the ICI-ITP phenomenon are all on the list of differential diagnoses, they may pose a challenge in selecting the optimal therapy for almost all individual cases. The history of immune checkpoint inhibitors’ first promising results dates back to the discovery of key inhibitory proteins in the late 1980s and 1990s [1]. Following this, the discovery of the CTLA-4 inhibitor and, later, PD-1 and its ligand, which restore or stimulate T-cell antitumour activity, became a landmark (honoured by the Nobel Prize) in modern oncology and oncohaematology, providing revolutionary results from 2011 to 2014 in many types and stages of cancer with previously poor or desperate prognosis [2,3]. Furthermore, LAG-3 is a co-inhibitory immune checkpoint protein on activated T cells and NK cells. Like PD-1 and CTLA-4, it acts as a “brake” on the immune system, preventing autoimmune reactions. It binds to MHC class II molecules on antigen-presenting cells and tumour cells, sending an inhibitory signal that suppresses T-cell proliferation, activation, and cytokine secretion. Tumours often exploit this checkpoint to evade immune detection, making it another important route for eliciting an antitumour response [4,5].

One of the most important haematological side effects of ICI, or ICI-containing therapeutic modalities, is ICI-induced thrombocytopenia (ICI-ITP). ICI-ITP is the main topic of this review, which highlights similarities and differences compared with traditional ITP [6,7]. On the other hand, platelet counts, activation, and PD-1 neoexpression on platelets might also play important roles in ICI efficacy. The pathogenesis of immune-related adverse events (irAEs) is also discussed in the second part.

## 2. Immune Checkpoint Inhibitor-Induced Immune Thrombocytopenia

ICI-ITP is a rare but serious complication characterised by a drop in platelets to <100 × 10^9^/L or less [8].

ICI-ITP affects approximately 0.25% of patients treated with ICIs and may lead to bleeding events and even increased mortality. The published incidence can be as high as 10%, depending on the tumour type and stage, and on the presence or absence and type of other chemo- or antitumour therapies. ICI-ITP may occur during treatment with all major classes of ICI agents, i.e., PD-1 and PD-L1 (common but later-onset) inhibitors, CTLA-4 inhibitors (early-onset but rare), and LAG-3 agents [7,8,9]. However, the frequency may vary within these subclasses across particular agents. Different cumulative incidence values also depend on the size of the patient population (0.1% comes from an 86,000-patient large-scale multicentre database). Still, there are many other factors, including geographical parameters, race differences, and heterogeneous medical care standards (early- or advanced-stage initiation treatments), combinations with or without myelosuppressive agents, neoadjuvant settings, the presence or absence of hormones (e.g., corticosteroids, hormone therapies), protocols for blood count checkups, and other yet-to-be-explored reasons.

**Figure 1:**
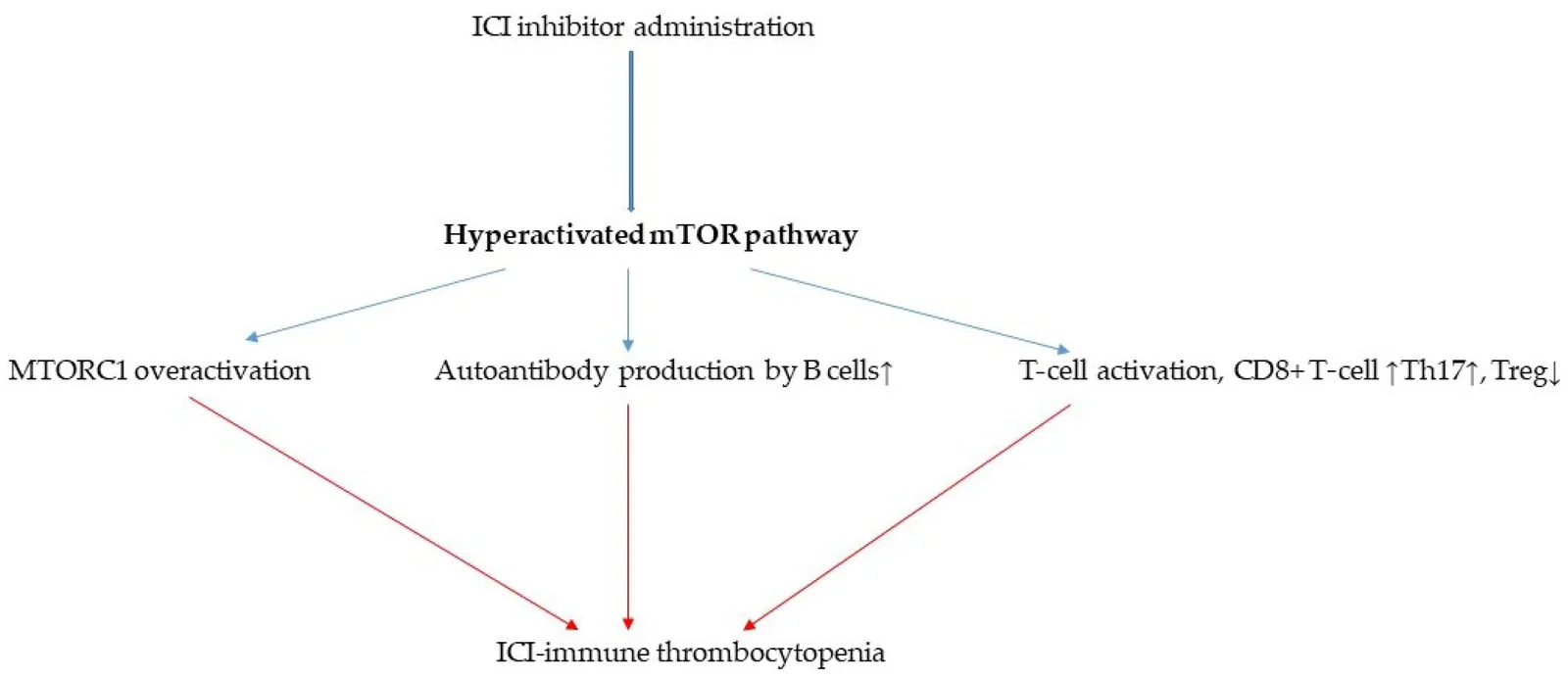
Hypothetical pathomechanism of ICI-ITP (not fully validated in human studies).

The chain of events in CD8+ and CD4+ T-cell activation, along with secondary mTOR dysregulatory events and interactions, is probably the most important common trigger, with different drugs having different chemical structures and cellular targets [7,10]. CD8+ cells and mTOR compounds can also impair megakaryocyte function [8,9]. Some elements and events of the complex, otherwise still hypothetical, pathomechanism are summarised in Figure 1.

### 2.1. Diagnosis and Differential Diagnosis

The differential diagnostic approach to immune thrombocytopenia conditions has become even more complex. When an ITP-like clinical condition develops, differentiating CVID based on detailed immunoglobulin G compartment analysis is an important diagnostic task. This is particularly relevant because CVID is associated with T-cell dysregulation and requires immunoglobulin replacement therapy, whereas ITP has relatively few specific laboratory diagnostic features apart from bone marrow megakaryocytosis.

The clinical background and history are usually, but not always, very unequivocal with ICI-ITP, but there are certainly some overlaps. Probably, the approach differs in each case, which might help to select the optimal therapy or, even more importantly, how to approach ICI-ITP prophylaxis. The detailed characteristics of classical chronic ITP and ICI-ITP are summarised briefly in Table 1.

The condition typically develops at a median of 8 weeks (range 4–18) after the first ICI administration, with a median nadir platelet count of 41 × 10^9^/L. Some cases (6–7% reported) are associated with easy bruising or, in some instances, more severe bleeding events (an average of 2–4%). ICI-ITP is associated with an increased risk of a fatal outcome, with a hazard ratio of 2.5–3.0 compared with patients without ICI-ITP [7,8,11]. The increased mortality may be explained by rare severe bleeding, corticosteroid administration (as a treatment for ICI-ITP), and, of course, discontinuation of ICI-based therapies [8]. Conversely, milder degrees of (non-ICI-ITP-type) thrombocytopenia (e.g., Grade 1) occasionally correlate with a stronger therapeutic response, suggesting heightened antitumor immunity [12,13,14,15].

### 2.2. Therapeutic Cornerstones

The standard therapeutic approach involves, in all grades and forms of ICI-ITP, first discontinuing (transiently or permanently) the ICI and initiating corticosteroids (e.g., prednisone 1 mg/kg). Approximately 50–60% of patients respond to this initial treatment within 2 weeks, but responses are usually not more durable than those in traditional chronic ITP. Thus, the general approach is to switch to other therapeutic modalities after 6–8 weeks. In the long run, immunosuppression might interfere with the efficiency of anticancer immunotherapy [12,14].

For non-responders or severe cases (grade 3 or 4, especially those at high bleeding risk), thrombopoietin receptor agonists (TPO-RAs) should be used, with response rates slightly above half the success rate as opposed to steroid-refractory classical ITP. Double-refractory cases are challenging and may require rituximab or high-dose intravenous immunoglobulin administration to improve platelet counts. Splenectomy might also be rarely considered, balancing benefits against the higher risk of infections in this endangered population [8,9,13,14].

Innovative medical approaches (rilzabrutinib, anti-CD38, mTOR modifiers, etc.) are essential approaches based on individual decisions made step by step. However, 30% of patients who undergo ICI re-exposure might experience recurrent thrombocytopenia [7,8,9,13]. Amegakaryocytic thrombocytopenia might also rarely occur [16].

Some interesting, exciting, and very promising new results are available on genetically modified, engineered platelets (megakaryocytes, and so on). The modified platelets are automatically transported via standard coagulation pathways to sites of vascular injury, such as surgical wounds or tumour vasculature. Engineered platelets are able to accumulate at surgical wounds and deliver chemotherapies in a site-oriented way, reducing general toxicity. They may deliver several clotting factors directly to the bleeding event in haemophiliacs. Some models are capable, quite importantly in our context, of accumulating immunocompetent cells in the tumour local microenvironment and also a special subset of engineered platelets that express PD-1. The latter may attract PD-L1 as a result of these interventions, targeting tumours by binding to PD-L1 on cancer cells, blocking immunosuppressive events, and inhibiting PD-L1 [17,18,19], but these remain pre-clinical findings.

In traditional ITP they may help with more efficient platelet substitution (IPSC-derived platelets) and transfer clotting factors to the bleeding site. These advantages might be useful in ICI-ITP for platelet supplementation and improve clotting and bleeding sites. PD-1-expressing variants of engineered platelets are useful tools to enhance local antitumour immune response [18,19].

## 3. ICI-ITP Prognostic Factors

### 3.1. Baseline Prognostic Elements

Some important baseline prognostic/risk factors should be evaluated very carefully in each case of candidates for ICI-based therapies. It would be desirable (with a standardised protocol) to try to predict the risk of post-ICI thrombocytopenia using these factors: a mild male preponderance, baseline thrombocytopenia per se or previous ITP, and potential others (congenital ADAMTS-13 deficiency, immunoglobulin deficiencies such as CVID/IgG2, presence of platelet surface immunoglobulins, granulated platelet count, coated platelets, and certain tumour types and treatment combinations; pembrolizumab cases require even greater attention regarding platelet counts [8,9,20,21,22,23,24]). ICI-ITP is more common in patients treated with PD-1/PD-L1 inhibitors [23]. It is not associated with the cancer type, line of therapy, or demographic factors.

### 3.2. Cell Counts and Ratios

Lymphopenia itself, or an increased neutrophil-to-lymphocyte ratio (NLR) over 3.7–5, might be considered indicative of a less-than-optimal ICI response, ICI-induced pneumonitis, pulmonary fibrosis, irAEs, and probably other immune complications, such as ICI-ITP. Pembrolizumab cases require even greater attention regarding platelet counts [10,14,22,24].

Interestingly, research indicates that patients with mild (e.g., Grade 1) baseline thrombocytopenia who receive ICI therapy may experience significantly improved overall survival compared with those who do not, potentially reflecting the role of platelet activation in more robust immune activation [25]. This association is particularly pronounced in non-small cell lung cancer, hepatocellular carcinoma, oesophageal carcinoma, and renal cell carcinoma, where a high platelet-to-lymphocyte ratio (PLR) reflects a chronic inflammatory and immunosuppressive tumour microenvironment that undermines ICI efficacy [10,11,24]. Additionally, higher baseline neutrophil counts and a higher NLR independently correlate with worse outcomes. At the same time, dynamic changes in these markers after the first treatment cycle further predict treatment response [15,24]. Eosinophilia is a strong predictor of irAEs and may thus be associated with a survival benefit with anti-PD-1/PD-L1 inhibitor treatment [26]. As a result, the platelet count and its ratio to lymphocytes are significant prognostic biomarkers in cancer patients receiving immune checkpoint inhibitors. Elevated baseline PLR is consistently associated with poor prognosis, including significantly shortened overall survival and reduced progression-free survival across multiple malignancies [17,18,19,27]. The NLR, PLR, and eosinophil counts are investigational observational surrogates, not validated biomarkers for routine clinical risk stratification.

### 3.3. Platelets

Platelets can promote tumour immune evasion and, along with a high platelet count, can trigger ICI-induced irAEs, including ICI-ITP. Severe ICI-ITP serves as a critical biomarker of broader pathologic immune activation and is associated with complications such as pneumonitis, pulmonary fibrosis, thrombotic events, and other irAE manifestations as listed above [9,10,21].

Platelets carry and release immunomodulatory molecules such as PD-L1, which can attenuate T-cell activity and promote immune tolerance within the tumour.

In rare cases (roughly one in 400 ICI patients), systemic immune activation leads to autoantibody-driven and T-cell-mediated platelet destruction, resulting in ICI-ITP [8,28].

ICIs can induce neutrophil–platelet aggregates and neutrophil extracellular traps, shifting the balance of haemostasis and potentially contributing to thrombotic adverse events as compared with patients with lower aggregate increments [8,9,10,20]. Research reveals that a decline in platelet levels—or severe cases of ICI-ITP—is independently linked to poorer survival outcomes and higher all-cause mortality, signifying that excessive systemic immune dysregulation has compromised the patient [25,29].

### 3.4. Immunoglobulins

Serum immunoglobulin levels, particularly those of IgG and IgA, serve as potential biomarkers for monitoring treatment response and predicting outcomes in cancer patients undergoing ICI therapy [30].

Beyond serum monitoring, immunoglobulins (particularly IgG and IgA) are frequently detected directly within various tumour tissues, including breast, lung, colon, and pancreatic cancers [1,27]. In these contexts, Ig expression is associated with tumour growth, invasion, metastasis, and immune escape, with high positive ratios observed in malignancies such as breast cancer (81% for IgG) and soft tissue tumours (97% for IgG) [31,32]. Monitoring serum immunoglobulins may help detect irAEs in patients with lung cancer. Studies indicate that gradual increases in serum IgG and IgA levels often precede the onset of irAEs during ICI treatment, suggesting that these changes can signal heightened immune responses before clinical symptoms appear [14,15,33]. Similarly to the PLR and eosinophil counts, serum immunoglobulin levels are not validated biomarkers.

### 3.5. Immune-Related Adverse Events

Platelet activation may trigger irAE development (i.e., coronary events) and sometimes may also accelerate it. It is probably a cause rather than a consequence [14,33]. In any case, it can be used as a marker for early detection or to assess the severity of irAEs.

Tumour ICI treatment-associated irAEs are mild-, moderate-, or high-risk complications, affecting many organs and biological functions. They might include ICI-ITP, which in turn may promote the development of other irAE manifestations [14,29,33].

The most important irAE endocrine manifestations are thyroiditis, thyroid or pituitary dysfunction, and type 1-like diabetes syndrome. Skin signs might also be present—mainly a rash, rarely vitiligo. Gastrointestinal signs are mostly diarrhoea and, less commonly, hepatic alterations. Pneumonitis, pulmonary fibrosis, myocarditis, pericarditis, and nephritis are dangerous irAE-induced complications. Neurological events span a broad range, including neuropathy, myasthenia, and motor and sensory dysfunction. Uveitis is fortunately uncommon [12,15,29].

## 4. Discussion

The restoration or amplification of host T lymphocytes, inducing an immune response against tumours by the so-called immune checkpoint inhibitor agents, such as CTLA-4 inhibitors, PD-1 inhibitors, and PD-L1 inhibitors, was and still is an undeniably huge step forward, providing unprecedented improvements in survival and mortality data, even in quite desperate cases. These agents can be administered alone, in combination, or, more frequently, sequentially, or sometimes simultaneously with chemotherapy or other biological therapies [1,2,3].

ICI-ITP is an extremely important complication, with potential bleeding complications, associated untoward immunological events, and postulated increased mortality and reduced survival. No doubt it is a shortcoming that many data come from anecdotal descriptions; even though multicentre trials were run, they examined a specified tumour type and therapeutic combination. This makes it necessary to synthesise non-homogeneous data and try to extrapolate general statements, considering the biases, and to avoid them as much as possible by focusing on common or overlapping features and clinical properties. Given that thrombocytopenia arising during treatment may not be exclusively attributable to immune checkpoint inhibitor therapy but may also result from bone marrow infiltration by the underlying malignancy, therapy-related myelodysplastic syndromes, or the effects of concomitant medications (including heparin-induced thrombocytopenia, antibiotics, chemotherapy, and other agents), these alternative aetiologies should be carefully considered to avoid the unnecessary discontinuation of ICI treatment. From a differential diagnostic perspective, a comprehensive review of concomitant medications, assessment of the temporal pattern of thrombocytopenia onset, and evaluation of the involvement of other hematopoietic cell lineages are essential. Although a characteristic temporal association with ICI exposure and the concurrent presence of other immune-related adverse events may favour a diagnosis of ICI-associated immune thrombocytopenia, exclusion of alternative causes remains necessary. Accordingly, the evaluation should include a peripheral blood smear examination and, when clinically indicated, a bone marrow assessment.

In ICI-ITP, ICI therapy is usually discontinued immediately if the thrombocytopenia is grade 3 or higher (platelets < 50 × 10^9^/L). ICI-ITP has a good average response to the suspension of ICI or standard medical treatment, with an overall response rate of approximately 75%, but approximately 30% of cases will return after the reinitiation of ICI, sometimes even with different ICI medication [11,13,25]. The side effects and combinations share many similarities across the inventory of ICIs; however, haematological sequelae and complications vary by agent, tumour type, therapeutic modality, and stage of cancer [7,34]. Cytopenic haematological events, including thrombocytopenia, might result from chemotherapy/biological treatments, from the ICIs themselves, or, more commonly, from both [7,8]. Despite this general approach, so-called ICI-ITP (platelet count falling below 100 G/L) develops relatively quickly (an average of 8 weeks after initiation of ICI-type therapies) in at least 0.25% of patients. It may cause easy bruising and bleeding, similarly to adult chronic classical ITP, and shares many other features of clinical presentation and the initial approach to medical treatment. However, the pathomechanism is quite different from that of ITP (T-cell activation plays a major role) and is associated with higher mortality than in non-ICI-ITP patients; in some circumstances, antitumour efficacy may also be worsened [10,11,13,24,25].

First- and second-line therapy-refractory ICI-ITP cases pose difficulties for further therapeutic approaches, which are similar, but not identical, to those in refractory chronic ITP [13].

The most common predictive factors associated with a greater likelihood of ICI-ITP are as follows: lower baseline platelet count, male gender (slightly), combination ICI therapy, and stage IV cancer [7,8,28,33]. The choice of agent seems to influence ICI-ITP, as observed with PD-1 and PD-1 ligand inhibitors, especially pembrolizumab, etc. [7,8,9,23].

### Clinical Dilemmas with ICI-ITP in Clinical Oncology and Oncohaematology

ICI-ITP is dangerous, with increased mortality from bleeding and tumour progression. The cessation of ICI or ICI-based therapy might pose a serious risk and disadvantage for many cancer or oncohaematology patients who badly need ICI component-containing therapeutic modalities to achieve prolonged remissions and overall survival [35]. This fact is very important: when further therapy seems necessary, and ICI-ITP is refractory, case-by-case decisions based on the risk/benefit ratio are made to determine whether to continue innovative treatments (see later) or to face the risk of splenectomy in this infection-endangered condition [34,36]. ICI-ITP and related immune complications, irAEs, seem largely independent of different indications. However, if a patient’s case requires an ICI or ICI-based therapeutic modality, male sex, prior thrombocytopenia, and the likelihood of chemo-provoked thrombocytopenia should also be part of individual decisions in such cases; standard selections and their biases are far from valid suggestions for most cases. Clearly, first- and second-line therapy for refractory ICI-ITP is very challenging. Still, in these cases, innovative therapies commonly used in refractory ITP should be considered, such as fosfatamatidine, rilzabrutinib, mycophenolate mofetil, azathioprine, and cyclosporine A or similar agents. TPO-RA agents might be especially useful if an ICI–chemo combination is administered, in which both arms of the therapy can induce thrombocytopenia, and both may respond to thrombopoietin stimulation. Even more difficult is the re-administration of ICI after ICI-ITP if there is no other choice to prolong patients’ lives, as only ICI therapy can achieve, or most probably achieve, this. Could ICI-ITP prevention work? Can we rely on that? Is there an established primary or secondary ICI-ITP prophylaxis suggested for clinical practice? Certainly, there are no double-blind, multicentre trials or guidelines/evidence available at present that could provide strong guidance on how to prevent ICI-ITP before the initiation of treatment. Secondary ICI-ITP’s incidence is 30%, and it typically develops after 8 weeks of re-administration of ICI. It occurs more frequently in patients with lower baseline platelet counts; or who are affected by previous autoimmune disease, had an immune adverse event after ICI, or have stage IV cancer; or for whom ICI is administered as part of a combination. This is a more complex situation in which you should or must re-administer an ICI-based or ICI-containing therapeutic protocol in a patient who had prior ICI-ITP. In that case, there is a chance of relapse after retreatment, but otherwise the oncological disease progression cannot be stopped. Thus, case reports on different prophylactic approaches are extremely important on this topic, and a probably somewhat empirical approach—what might be considered in these conditions necessitating ICI-ITP prevention—can also be important.

Primary prophylaxis means careful risk assessment and preselection. In higher-primary-risk patients receiving ICI–chemo, applying TPO-RA is probably useful [23]. For secondary prophylaxis, it seems likely that steroids before re-administration should be considered by individual risk–benefit assessment (side effects, immunosuppression, and tumour progression should be evaluated on an individual basis). An innovative approach would be an mTOR inhibitor or an anti-CD38 monoclonal antibody; daratumumab might also be considered [34,36]. Prophylactic splenectomy might carry the risk of more infections (which may be mitigated by standard splenectomy vaccination), especially if any evidence of increased spleen size can be detected [34,35,36]. As ICI administration may provoke thromboembolic events, the use of some anticoagulant prophylaxis is broadly recommended, but it certainly has to be stopped when the platelet count is less than 50 G/L [35,37]. As part of secondary prophylaxis, this should be discontinued or thoroughly checked in ICI-ITP-treated patients, or in those who need ICI-ITP prophylaxis. Screening for thrombotic microangiopathy (antibody-induced type) might also be warranted, and plasma exchange and plasmapheresis might be an option in this case [17,35]. On the other hand, a slightly lower baseline platelet count is associated with better outcomes in cancer patients with ICI-based therapies; local platelet activation may play an important role in tumour progression, while elevated baseline platelet values seem to be associated with less efficient tumour treatment [2,28].

## 5. Conclusions

ICIs have revolutionised cancer treatment, but they can also cause irAEs such as ICI-ITP. Early recognition, differential diagnosis, and well-tailored individual management—including standard and, if necessary, innovative elements of treatment—are all extremely important. Even though official guidelines are not available for primary or relapsed case prophylaxis, it is essential to find individual approaches to it. It is essential to balance patient safety with the benefits of cancer immunotherapy. It might be more difficult to establish well-standardised multicentre controlled trials in this very high-risk and heterogeneous patient cohort; the step forward in this direction seems crucially important.

## Figures and Tables

**Table 1:** Comparison of classical versus ICI-ITP.

Features	Classical ITP	ICI-ITP
Background	The chronic adult form is typically idiopathic, though it is sometimes associated with IgG subclass deficiencies.	Starts as an immune-related adverse event (irAE) during cancer therapies involving ICI modalities.
Timing properties	Difficult to establish: chronic, undetermined, subtle.	Relatively quick, with a similar onset at a median of 8 weeks during ICI therapy.
Pathological events	Loose antibody binding to the platelet surface, sometimes provoked by T-cell abnormalities and increased platelet sequestration.	Global, more general immune hyperfunction: a T-cell-mediated response against self-antigens, including platelet structures.
Risk factors	Often unknown; associated with certain viral infections or autoimmune predispositions.	Previous or pretreatment thrombocytopenia, advanced cancer, other irAEs, etc.
Mortality issues	Thrombocytopenia can be treated effectively, and mortality is low.	In almost all cases of severe thrombocytopenia, there is an approximately 2.5- to 3-fold higher risk of death, and sometimes worse oncological outcomes (but higher mortality in ICI-ITP may reflect cancer severity, treatment interruption, bleeding, steroid exposure, systemic immune dysregulation, etc.)
Recommended interventions	Responds to a well-known sequence of steroids, intravenous Ig, and TPO-RAs.	Stop ICI administration, either temporarily or permanently; treat with ITP-type drugs if needed (steroids, TPO-RA, refractory intravenous Ig or rituximab). Thirty percent of patients develop a recurrence if rechallenged with ICI therapy.

## Data Availability

No new data were created or analyzed in this study. Data sharing is not applicable to this article.

## Abbreviations

ADAMTS-13	A disintegrin and metalloprotease with thrombospondin type 1 motif; lack or inhibition causes thrombotic microangiopathy
CVID	Common variable immune deficiency
ICI	Immune checkpoint inhibitor
ICI-ITP	Checkpoint inhibitor-associated thrombocytopenia
IgG	Immunoglobulin G
ITP	Immune (idiopathic) thrombocytopenic purpura
IRAE	Immune-related adverse event
CTLA-4 inhibitor	Inhibitor of Cytotoxic T-lymphocyte-associated protein 4
LAG-3	Lymphocyte activation gene-3
MHC	Major histocompatibility complex
NLR	Neutrophil–lymphocyte ratio
mTOR	Mammalian target of rapamycin, immune and metabolism regulatory serine protease
PD-1	Programmed cell death protein 1
PD-L1	Programmed cell death ligand 1
PLR	Platelet–lymphocyte ratio
TPO-RA	Thrombopoietin receptor agonist
